# Rethinking Cell Phone Use While Driving: Isolated Risk Behavior or a Pattern of Risk-Taking Associated with Impulsivity in Young Drivers?

**DOI:** 10.3390/ijerph18115640

**Published:** 2021-05-25

**Authors:** Elizabeth A. Walshe, Flaura K. Winston, Dan Romer

**Affiliations:** 1 Center for Injury Research and Prevention, The Children’s Hospital of Philadelphia, Philadelphia, PA 19104, USA; winston@chop.edu; 2 Annenberg Public Policy Center, University of Pennsylvania, Philadelphia, PA 19104, USA; dan.romer@appc.upenn.edu

**Keywords:** young drivers, motor vehicle crashes, cell phone use, impulsivity, risky driving practices

## Abstract

This study examines whether cell phone use stands apart from a general pattern of risky driving practices associated with crashes and impulsivity-related personality traits in young drivers. A retrospective online survey study recruited 384 young drivers from across the United States using Amazon’s Mechanical Turk to complete a survey measuring risky driving practices (including cell phone use), history of crashes, and impulsivity-related personality traits. Almost half (44.5%) of the drivers reported being involved in at least one crash, and the majority engaged in cell phone use while driving (up to 73%). Factor analysis and structural equation modeling found that cell phone use loaded highly on a latent factor with other risky driving practices that were associated with prior crashes (*b* = 0.15, [95% CI: 0.01, 0.29]). There was also an indirect relationship between one form of impulsivity and crashes through risky driving (*b* = 0.127, [95% CI: 0.01, 0.30]). Additional analyses did not find an independent contribution to crashes for frequent cell phone use. These results suggest a pattern of risky driving practices associated with impulsivity in young drivers, indicating the benefit of exploring a more comprehensive safe driving strategy that includes the avoidance of cell phone use as well as other risky practices, particularly for young drivers with greater impulsive tendencies.

## 1. Introduction

In North America, both epidemiological and observational studies have shown that cell phone use while driving is associated with increased crash and near-crash risk [1,2]. In 2018, distracted driver crashes accounted for 8% of fatal crashes on U.S. roads, killing 2841 people [3]. Furthermore, compared to all other age groups, young adults aged 20–29 years are over-represented in cell phone-related fatal crashes [3]. Currently, 20 states and the District of Columbia (D.C.) have bans on hand-held phone conversations for all drivers, and 48 states have specific texting bans [4]. For young drivers, specifically, 38 states and D.C. have cell phone bans. Evaluations of the effectiveness of these interventions show mixed results: some evidence reductions in cell phone use [5,6]—and perhaps more so for handheld calls than texting bans—but inconsistent evidence of reductions in motor vehicle crashes [7]. Although the success of these policies may have been limited by insufficient education and enforcement, an insurance claims analysis showed that cell phone bans were not effective in reducing crashes, even in areas with high-visibility enforcement [8]. 

One possibility is that drivers who frequently engage in cell phone use while driving may also engage in other intentional risky practices while driving [9]. A 2013 study of Boston-area drivers aged 20–69 years found that self-reported cell phone use while driving was associated with more self-reported risky driving behaviors and observed risky driving during an on-road assessment [10]. A 2018 study of Philadelphia drivers aged 18–20 years found a similar pattern of self-reported risk [11]. In other words, while cell phone use may be one source of risk, this behavior may reflect a larger propensity to engage in a variety of risky driving activities. These data suggest that cell phone use while driving may not adequately address the underlying risk, which may highlight a need for interventions addressing patterns of risky practice (rather than targeting the individual behavior of cell phone use) [12].

There is also evidence of a link between impulsivity-related personality traits, specifically, weaker impulse control (acting without considering consequences) and sensation seeking (seeking novel and exciting experiences) and risky driving practices, including cell phone use, in young drivers [13,14]. In addition, weakened ability to delay the gratification of rewards, considered a behavioral indication of weak impulse control, has been positively associated with cell phone use on the road in some studies [15,16], but not in another [17]. One study found that impulsivity also partially explained differences in driving performance between recreational cannabis users and controls [18]. Drivers who are more impulsive may find it difficult to ignore cell phone alerts while driving and avoid other risky driving practices, and those with high sensation seeking tendencies may be more inclined to take other risks on the road, such as running red lights or driving after consuming alcohol [19]. 

There are well-documented developmental changes in impulsivity-related personality traits that may explain increased risk-taking behaviors in young drivers [20,21,22,23]. Neuro-behavioral theories suggest that this is attributable to ongoing structural and functional changes in the brain (through adolescence and into adulthood). In particular, the ongoing maturation of the prefrontal cortex and the associated executive control system that supports self-regulation and cognitive control over behavior may explain weaker impulse control, while rising dopaminergic activity in the brain’s reward circuit may also explain increased sensation seeking [20,21,22,23,24,25,26]. However, there is also evidence for substantial individual variability in these traits and the underlying neural and cognitive development, which may explain variable risk-taking in young drivers [24]. If impulsive personality differences underlie tendencies to engage in risky driving practices, including cell phone use, then interventions that discourage the use of cell phones with respect to underlying personality differences may be more effective.

In order to better understand risky driving practices, the current study recruited a sample of young adult drivers from across the United States to determine: (i) whether cell phone use while driving is only one of several other co-occurring risky driving practices in which young drivers engage (replicating prior findings); (ii) if this pattern of dangerous driving behavior is related to crashes (maintaining constant factors such as sex and number of years driving); and (iii) what role sensation seeking and impulsivity play in the relationship between risky driving and crashes. Thus, this study expands on our prior work by including a larger and more geographically diverse sample, with the addition of other known risk factors of impulsivity-related personality traits. We hypothesize that (i) drivers who frequently engage in cell-phone use while driving will also frequently engage in other risky driving behaviors associated with crashes, and (ii) that this pattern of risk-taking will be associated with weaker impulse control (due to the ongoing maturation of executive cognitive control at the transition to adulthood). If a pattern of risky driving is associated with a history of crashes and risk-taking personality differences, then this has important implications for future research to inform the design and delivery of interventions at both the individual and public health level, potentially calling for a more personalized prevention strategy.

## 2. Materials and Methods

### 2.1. Sample

Participants were recruited via the Amazon MTurk Prime Panels and directed to an online survey hosted by the Psytoolkit survey platform. A sample of 384 licensed drivers aged 18–24 years from across the United States completed the survey, and all data were collected over the course of 5 days in November 2017: the mean age was 21.41 years (SD = 1.96). Table 1 shows the sample demographics. Drivers participated from across the four U.S. census regions (including 44 states: see Table 1). Two participants reported having a license from another country. The University of Pennsylvania Institutional Review Board (IRB) determined this study exempt from its review because no sensitive or identifiable information was collected from survey respondents.

### 2.2. Survey

The survey consisted of questions capturing self-reported crash and citation history and risk-related driving practices. In addition, 3 quality assurance questions were included that asked participants to respond in a specific way in order to measure attention to the survey (e.g., “Please select “No” below, for quality control purposes”). If two questions were answered incorrectly, that participant’s data would be removed from analyses. No participants were excluded from the analysis for lack of attention. 

History of crashes was recorded as in our prior work (response options: “none”, “one”, “two”, “more than two”) [11]. Additionally, replicating our prior studies [11,24], a subset of DBQ survey items that were deemed to pose “definite risk to others’’ was included from the Driver Behavior Questionnaire (from the two most commonly reported latent constructs: deliberate behavioral violations and driving errors) [27] with two additional items that asked about answering a text while driving and talking on a cell phone while driving. For all driving behavior items, participants indicated how often they engaged in each on a 6-point scale (1 = never, to 6 = nearly all the time). We also assessed another risky practice from the Youth Risk Behavior Survey [9]: non-use of seat belts as a passenger or driver.

The survey also included self-report measures of sensation seeking and acting-without-thinking, a form of impulsivity. Sensation seeking was measured with 4 items from the Brief Sensation Seeking Scale [28], with responses ranging from 1 = strongly disagree to 4 = strongly agree (Cronbach α = 0.80). (2) Acting-without-thinking was measured with 6 items from the Junior Eysenck Impulsivity Scale [29], coded as 0 or 1, where 1 = impulsivity (Cronbach α = 0.80). Another form of impulsivity, known as delay discounting—the ability to delay immediate rewards—was measured by an adapted monetary choice task [30]. (These personality scales are described in more detail in prior work: [20,25]).

### 2.3. Statistical Analyses

Correlations between each risky driving practice and crashes were examined, along with other variables. A confirmatory factor analysis was used to test a two-factor versus a single-factor structure of risky driving practices identified in a prior sample [11]. First, we explored the relationship between risky driving, crashes, and both forms of impulsivity-related traits: structural equation modeling was used to regress the binary crash outcome on risky driving (including cell phone use) identified in the factor analysis and to determine whether those practices were associated with either sensation seeking or impulsivity. Variables that were not associated with crashes in preliminary model testing were dropped in subsequent models (including U.S. region). Secondly, to further test the role of cell phone use alone or combined with other risky driving practices, we removed cell phone use from the risky driving score and created three dummy variables representing people: (1) high on risky driving practices and low on cell phone use; (2) low on risky driving practices and high on cell phone use; (3) and high on both risky driving practices and cell phone use. Those who scored low on both factors were the reference group. For all models, standard goodness of fit indices were used. All analyses and models were computed in Mplus version 8 (Muthén and Muthén).

## 3. Results

### 3.1. Driving History and Behavior

On average, this sample became licensed at age 17.2 years (SD = 1.7), and at the time of the study, had been licensed for 4.2 years (SD = 2.3; range: 0–9 years). Among the 384 drivers, 171 (44.5%) reported being involved in at least one crash as a driver. Of the 171 who reported a crash history, 46 (27%) drivers had two prior crashes, and 18 (11%) drivers had more than two prior crashes. While these multiple crash drivers reported more engagement in risky driving behaviors, they were not significantly different from drivers who reported one prior crash, therefore we collapsed these groups to form a binary crash history outcome for simplicity in our models. The majority of participants (*n* = 281, 73.2%) reported engaging in cell phone calls while driving, and 237 (61.7%) reported answering a text message while driving. Several of the behaviors were related to experiencing a prior crash apart from cell-phone use (see Table 2). The two cell phone items were highly correlated (*r* = 0.713, *p* < 0.001). In other aspects of risk-taking, 15 drivers (3.9%) reported never or rarely wearing a seat belt as a passenger, and 7 (1.8%) reported never or rarely wearing a seat belt as a driver, which is comparatively lower reported risk-taking with regard to seatbelt use compared to national observations on these same survey items [9,31]. Of note, some of the individual behaviors, including cell phone use, had statistically significant correlations with prior crashes. Although these significant correlations were small, the cell phone use items along with driving after consuming alcohol were among the highest.

### 3.2. Latent Structure of Dangerous Driving Practices

The factor analysis confirmed a correlated two-factor model, as previously identified [11]. Items 1–4, 9, and 10 loaded on the first factor, with items 5–8 loading on a second factor, and the data fit this model well: CFI = 0.976; TLI = 0.964; and RMSEA = 0.054. The data fitted better when including Mplus-suggested modifications to account for additional covariance between items 2 and 3, 6 and 7, 7 and 8, and 9 and 10. However, the two factors were highly correlated (*r* = 0.933), and a single-factor model also showed good fit for the data: CFI = 0.971; TLI = 0.958; and RMSEA = 0.058. Table 3 shows the standardized item loadings for the single factor, which was used in further analyses. An observed score derived from the standardized values of the items, which we termed “risky driving” practices, showed high internal consistency (Cronbach α = 0.86).

### 3.3. Associations with Crashes

Table 4 provides the correlation coefficients between crashes and the individual characteristics and dangerous driving practices, as well as the factor score described above. As expected, age, sex, and number of years licensed were associated with crashes. Not surprisingly, age and number of years licensed were also highly correlated (*r* = 0.7). Sensation seeking and the risky driving factor were also positively correlated with crashes, while acting-without-thinking was only correlated with risky driving (along with the number of years licensed and sensation seeking). Delay discounting was not associated with crashes or risky driving. Of note, although the correlations between the individual risky driving survey items and crashes presented above in Table 1 were sometimes low (however significant at the *p* < 0.01 level), the combined risky driving factor was also significantly associated with crashes. A structural equation model was tested that regressed the binary prior crash variable on the risky driving factor, sensation seeking, and acting-without-thinking, while controlling for sex and number of years licensed. The model (Crash Model 1) fit the data well (CFI = 0.92; TLI = 0.91; and RMSEA = 0.04 [90% CI, 0.03–0.05]). Crashes were significantly associated with risky driving (*b* = 0.16, SE = 0.07, *p* = 0.036 [95% CI: 0.019, 0.306]) and sensation seeking (*b* = 0.48, SE = 0.16, *p* = 0.004 [95% CI: 0.177, 0.837]), but not acting-without-thinking (*b* = −0.09, SE = 0.26, *p* = 0.740).

**Figure 1:**
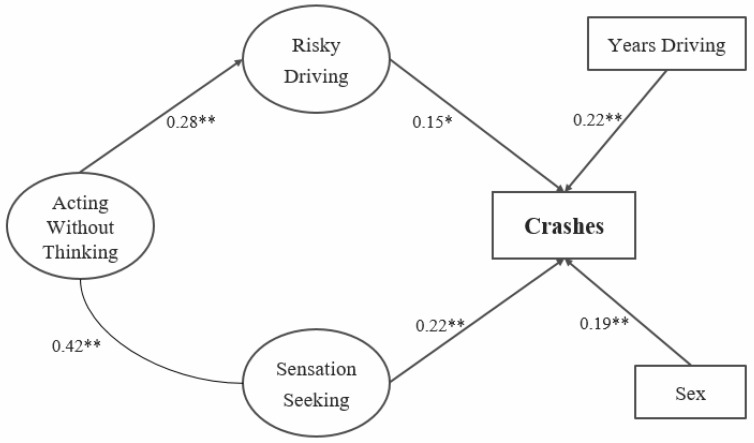
Path diagram for Crash Model 2 illustrating the association between risky driving (including cell phone use), sensation seeking, and crashes. Note: straight lines indicate direct regression paths, with standardized beta values in the SEM model. The curved connector indicates the standardized estimate of correlation between variables. * indicates significance at the *p* < 0.05 significance level, with ** representing significance at the *p* < 0.01 level.

The model was modified to test for any indirect effect of acting-without-thinking on crashes through risky driving (using 1000 bootstrap samples), while removing the direct relationship between acting-without-thinking and crashes (Crash Model 2). This model fit the data well (CFI = 0.93; TLI = 0.92; and RMSEA = 0.04 [90% CI, 0.03–0.04]). Figure 1 shows the Crash Model 2 path diagram. The relationship between crashes and risky driving (*b* = 0.15, [95% CI: 0.01, 0.29]) and sensation seeking (*b* = 0.44, [95% CI: 0.16, 0.76]) remained while controlling for the other variables. In addition, risky driving was related to acting-without-thinking (*b* = 0.84, [95% CI: 0.38, 1.45]). There was an indirect relationship between acting-without-thinking and crashes through risky driving (*b* = 0.127, [95% CI: 0.01, 0.30]), indicating that persons engaging in risky driving were also more likely to act without thinking.

**Figure 2:**
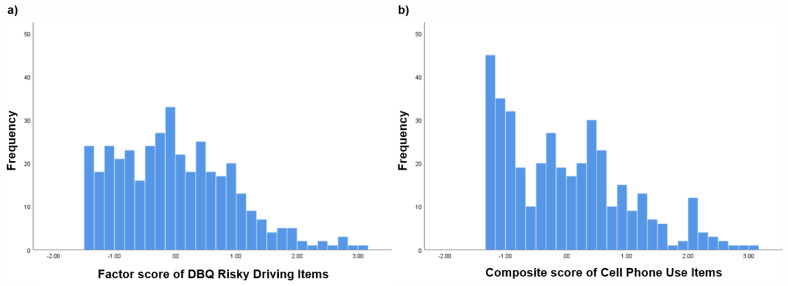
Frequency distribution of responses on (**a**) risky driving items, and (**b**) cell phone use items.

Although cell phone use loaded on the risky-driving factor, we aimed to determine whether cell phone use alone apart from the other behaviors on the factor was associated with prior crashes. We created three risk-level indicators based on a median-split of risk-factor scores after removing cell phone use from the score (see Figure 2 for a distribution of behavior scores before the median split): (1) high on risky driving and low on cell phone use; (2) low on risky driving and high on cell phone use; and (3) high on both. Other variables included in the model were sensation seeking, sex, and years driving. Indicators 1 and 2 were not associated with crashes and were thus dropped from the model. The resultant model provided a good fit for the data (Crash Model 3): CFI = 0.96 TLI = 0.94; and RMSEA = 0.04 [90% CI, 0.00, 0.06]. Crashes were still significantly related to sensation seeking (*b* = 0.45, [95% CI: 0.13, 0.39] and the indicator representing high risk on both risky driving and cell phone use (*b* = 0.29, [95% CI: 0.02, 0.26], while controlling for the effects of sex and years driving.

## 4. Discussion

This study found that cell phone use while driving was only one indicator of a more general pattern of risky driving practices which is associated with prior crashes in young adult U.S. drivers. In addition, risky driving was associated with the form of impulsivity known as acting-without-thinking. Sensation seeking was also associated with crashes, but independently of risky driving practices and impulsivity. These data also showed that drivers who reported engaging in cell phone use while driving also reported engaging in other risky behaviors, such as ignoring speed limits, dangerous overtaking, and taking chances going through red traffic lights. Frequent cell phone use while driving by itself (apart from other risky driving practices) was not associated with prior crashes. These findings support the hypothesis that cell phone use while driving is but one of several risky driving practices in young people that may contribute to crashes.

These results replicate those of [11], and corroborate prior studies suggesting patterns of risky practices among drivers, which are related to individual characteristics [9,10]. However, this study expands upon prior work by recruiting a larger sample of young adult drivers to include some representation from the four U.S. census regions (making these findings more generalizable, although not nationally representative), and with the addition of multiple measures of impulsivity-related personality traits that are associated with risk-taking in young drivers. This study found that the relationship between risky driving and crashes was attributable in part to higher levels of one type of impulsivity, acting-without-thinking. This finding supports research that has linked risky driving practices, and specifically cell phone use while driving, to weak impulse control [13,17]. Sensation seeking was also associated with crashes, but not as mediated by risky driving practices. Prior work has shown that it is associated with a potentially different pattern of risk behaviors, including substance use and driving with peers [19,32], that may explain its relationship with crashes. Future work should examine the role of substance use and also executive function in dangerous driving and crashes. While sensation seeking as assessed by self-report overlaps with acting-without-thinking, these tendencies are separable and show different developmental trajectories across adolescence and different relationships with higher-order executive functions [20,21,22] and risky driving (including cell phone use) [13,17]. Thus, these traits may represent an important avenue for future study to delineate the underlying neurocognitive risk for crashes in young drivers.

Taking these two findings together, future research could guide interventions that address the driver rather than the behavior. For example, when drivers are identified as at-risk based on individual characteristics (e.g., diagnoses associated with high impulsivity such as ADHD) or behaviors (e.g., citations or crashes for risky driving practices), the avoidance of cell phone use as well as other risky practices could be included in driving countermeasures. Clinic-based brief interventions could expand the model developed for targeting young drivers who present with injuries after alcohol-related crashes [33,34]. These clinical interventions could address not only alcohol use but also engagement in a range of related dangerous driving practices, including, but not limited to, cell phone use while driving. This will require research to identify effective messages to convey the risk that these behaviors create.

### Limitations

The driving practices and crash outcomes in the current (and prior) study relied on self-report responses. However, the risk of social desirability bias may have been reduced by the anonymous recruitment of participants and online survey. Furthermore, prior work has also relied on self-reported crashes (which can be over- or under-reported) [35,36,37], including models with a history of crashes as the outcome [24,38]. While naturalistic studies and in-vehicle monitoring can offer prospective crash monitoring, and provide richer information about crashes (e.g., if the driver was at fault), these approaches are not without limitations (e.g., recruitment bias and crashes are often a rare outcome of such studies). Survey studies still provide many advantages for data collection (more diverse samples, more history of crashes). This retrospective study design can also mean that the crashes reported could be from any point before the time of this survey (early in licensure or later). However, the number of years driving was controlled for in the analysis. We used a binary crash outcome that collapsed multiple-crash drivers into one group of “at least 1 crash” (described above in our results). This allows us to replicate our prior work and that of others [11,24,38], but future work should explore multiple crashes as an alternative outcome because these drivers may be more at risk. Furthermore, while the measures of personality traits were taken after the driver’s had a crash, these traits are largely stable across age in young adulthood [39], allowing us to examine the pattern of risk taking. However, future studies should confirm the findings through a prospective longitudinal study.

This study relied on Mechanical Turk as a recruitment method, which might be subject to recruitment bias; however, there is evidence to suggest that MTurk samples are more diverse than other online survey samples and American campus samples [40]. Furthermore, while this sample can be considered more generalizable than the prior study conducted with Philadelphia drivers, and the study participants reported seat-belt use comparable to nationally representative samples, the sample was not, in fact, nationally representative, nor did it examine differences between rural- and urban-based drivers. These factors should be considered in future work.

A further limitation of this work is that the characterization of cell phone engagement was limited and did not specify if the call was hand-held or not, nor did we ask about the use of any other cell phone features while driving (e.g., browsing), or other forms of distracted driving (e.g., passenger-related). In addition, trends in phone use while driving among young drivers may have changed since these data were collected in 2017. Future work should consider including more items addressing contemporary risky driving practices to capture cell phone distractions while driving.

## 5. Conclusions

These findings support prior research to suggest that cell phone use while driving is part of a pattern of risky driving associated with crashes that is also associated in part with differences in acting-without-thinking impulsivity. Furthermore, current point-of-care brief interventions (e.g., for alcohol-impaired driving) could consider broadening their scope from addressing one risky behavior. When these drivers are identified as engaging in any one of these dangerous practices, there is an opportunity to deliver interventions that also address related risk behaviors when being treated for injuries after a crash or when pulled over following a citation for dangerous behavior. Assessments of personality traits related to crashes may also help in the early identification of drivers more at risk, who may stand to benefit most from targeted interventions.

## Figures and Tables

**Table 1:** Sample characteristics: gender, age, and U.S. census region.

Characteristic	*n*	%
Gender		
Male	216	56.30%
Female	166	43.20%
Transgender/Other	2	0.50%
Age		
18 years	37	9.60%
19 years	45	11.70%
20 years	50	13.00%
21 years	55	14.30%
22 years	55	14.30%
23 years	73	19.00%
24 years	69	18.00%
Census Region		
Northeast	66	17.19%
Midwest	100	26.03%
South	140	36.44%
West	76	19.78%

**Table 2:** Risky driving behavior frequencies and correlations with crashes. Note: ** = statistically significant correlation with crashes where *p* < 0.01.

Driving Survey Items	Never	Hardly Ever	Occasionally	Quite Often	Frequently	Nearly All the Time	Relation to Crashes
Ignored speed limits late at night or early in the morning?	26.82% (*n* = 103)	19.79% (*n* = 76)	28.39% (*n* = 109)	10.94% (*n* = 42)	10.68% (*n* = 41)	3.39% (*n* = 13)	*r* = 0.058
Drove close to a car ahead of you or flashed your lights as a signal to go faster or get out of your way?	52.60% (*n* = 203)	21.61% (*n* = 83)	15.10% (*n* = 58)	7.81% (*n* = 30)	8.02% (*n* = 8)	0.78% (*n* = 3)	*r* = 0.062 **
Became impatient with a slow driver in the left passing lane and passed on the right?	22.40% (*n* = 86)	19.97% (*n* = 69)	29.95% (*n* = 115)	15.10% (*n* = 58)	11.46% (*n* = 44)	3.13% (*n* = 12)	*r* = 0.092
Drove with only “half an eye” on the road while looking at a map or using the controls in the car?	30.21% (*n* = 116)	21.09% (*n* = 81)	30.47% (*n* = 117)	11.98% (*n* = 46)	5.21% (*n* = 20)	1.04% (*n* = 4)	*r* = 0.099 **
Took a chance on going through an intersection when the light turned red?	56.51% (*n* = 218)	22.66% (*n* = 87)	14.32% (*n* = 55)	2.34% (*n* = 9)	4.17% (*n* = 16)	0.00% (*n* = 0)	*r* = 0.07
Drove after consuming alcohol?	76.30% (*n* = 294)	18.49% (*n* = 71)	3.39% (*n* = 13)	1.04% (*n* = 4)	0.52% (*n* = 2)	0.26% (*n* = 1)	*r* = 0.191 **
Misjudged the speed of an oncoming vehicle when passing a car?	52.34% (*n* = 202)	29.43% (*n* = 113)	14.58% (*n* = 56)	2.34% (*n* = 9)	1.04% (*n* = 4)	0.26% (*n* = 1)	*r* = 0.079
Failed to check your mirrors before pulling out of a parking spot or changing lanes?	43.23% (*n* = 166)	34.64% (*n* = 133)	16.67% (*n* = 64)	3.65% (*n* = 14)	1.04% (*n* = 4)	0.78% (*n* = 3)	*r* = 0.031
Talked on a cell phone while driving?	26.82% (*n* = 103)	22.66% (*n* = 87)	25.78% (*n* = 99)	11.72% (*n* = 45)	11.98% (*n* = 46)	1.04% (*n* = 4)	*r* = 0.223 **
Answered a text message while driving?	38.28% (*n* = 147)	22.66% (*n* = 87)	23.43% (*n* = 90)	8.33% (*n* = 32)	6.51% (*n* = 25)	0.78% (*n* = 3)	*r* = 0.167 **

**Table 3:** Standardized item loadings on the single factor structure of risky driving practices.

Item	Driving Survey Item	“Risky Driving”
4	Drove with only “half an eye” on the road while looking at a map or using the controls in the car?	0.76
1	Ignored speed limits late at night or early in the morning?	0.71
10 ⁺	Answered a text message while driving?	0.68
3	Became impatient with a slow driver in the left passing lane and passed on the right?	0.65
2	Drove close to a car ahead of you or flashed your lights as a signal to go faster or get out of your way?	0.64
9 ⁺	Talked on a cell phone while driving?	0.59
5	Took a chance on going through an intersection when the light turned red?	0.58
8	Failed to check your mirrors before pulling out of a parking spot or changing lanes?	0.47
6	Drove after consuming alcohol?	0.45
7	Misjudged the speed of an oncoming vehicle when passing a car?	0.39

⁺ Indicates cell phone items that are not part of the traditional Driver Behavior Questionnaire.

**Table 4:** Table of Pearson’s correlation coefficients between variables.

	Correlation Coefficient						
Variable	1	2	3	4	5	6	7
1. Age							
2. Sex	0.063	-					
3. Years driving	0.700 **	0.122 *	-				
4. Sensation Seeking	−0.081	−0.178 **	−0.045	-			
5. Acting-Without-Thinking	−0.186 **	−0.059	−0.075	0.478 **	-		
6. Delay discounting: lowest amount	−0.088	0.105 *	0.007	0.056	−0.043	-	
7. Risky Driving Factor	0.027	0.012	0.173 **	0.244 **	0.334 **	0.086	-
8. Crashes	0.180 **	0.135 **	0.204 **	0.163 **	0.079	−0.017	0.168 **

* 0.05 significance level (2-tailed); ** 0.01 significance level (2-tailed); Note: Risky Driving Factor includes cell phone use items.

## Data Availability

The data presented in this study are available on request and upon signing a data use agreement from author Dan Romer.
